# Supplementary material for: Distinct fate, dynamics and niches of renal macrophages of bone marrow or embryonic origins
Source: Nat Commun. 2020 May 8;11:2280. doi: 10.1038/s41467-020-16158-z (PMC7210253; doi:10.1038/s41467-020-16158-z)
Supplement: Supplementary file 1 — Supplementary Information [file 41467_2020_16158_MOESM1_ESM.pdf]

Supplementary Information

Distinct fate, dynamics and niches of renal macrophages of bone marrow or embryonic origins

Liu et al.

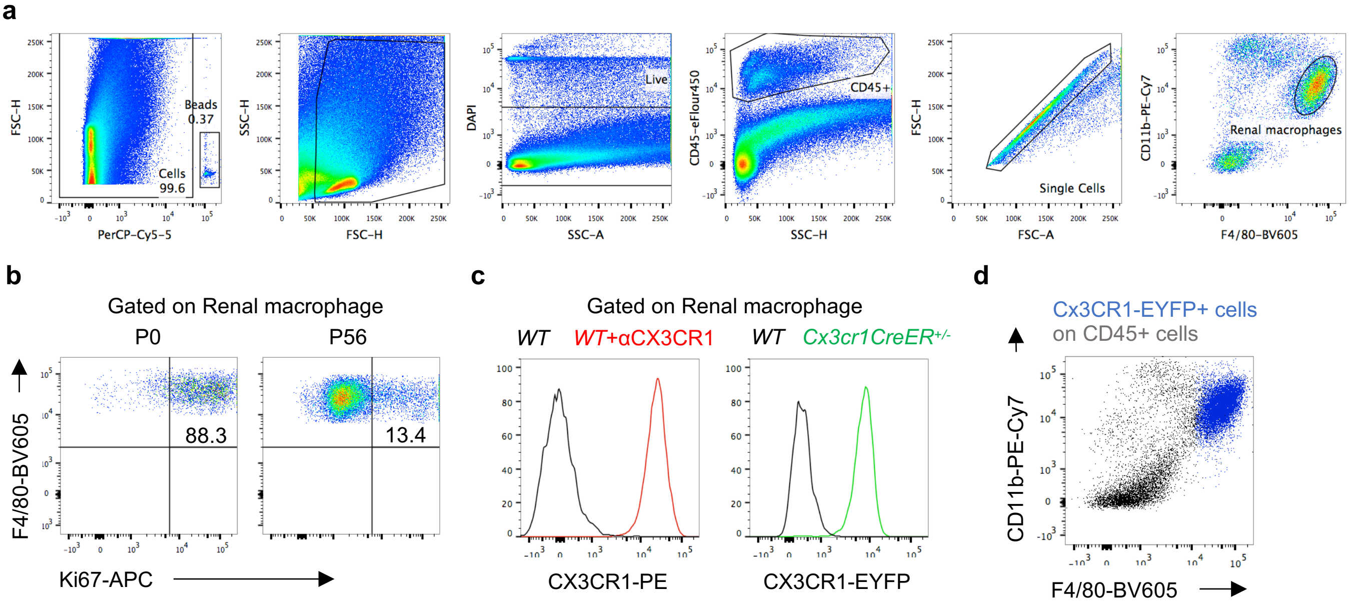

Supplementary Figure 1. Flow cytometry of Renal macrophages (RMs). (**a**) Gating strategy of F4/80^{high} RMs. (**b**) Ki67 staining of RMs at newborn (P0) and adult (P56) mice. (**c**) Histogram of CX3CR1 expression in RMs from *WT* and *Cx3Cr1CreER-EYFP* mice. (left: RMs from C57 WT mice were stained with isotype-PE or αCX3CR1-PE antibody; right: EYFP expression in RMs from C57 WT mice and *Cx3Cr1CreER*$^{+/-}$ mice).(**d**) CX3CR1-EYFP was exclusively expressed in F4/80^{high} RMs.

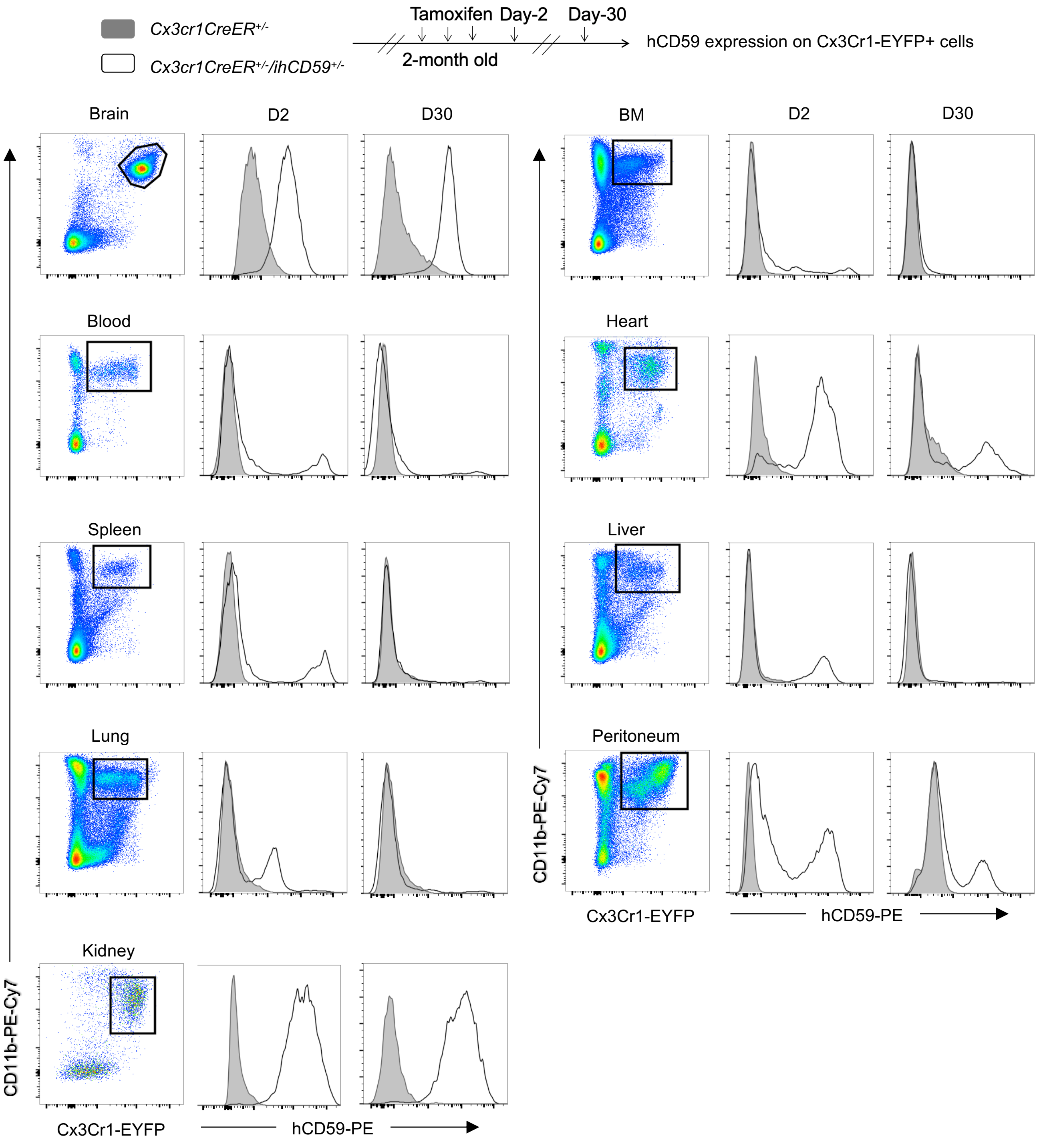

Supplementary Figure 2. Tamoxifen-induced hCD59 expression in different tissue. hCD59 expression in CD11b+CX3CR1+ cells in different tissues at 2 days (D2) and 30 days (D30) after Tamoxifen injection. Dot plots show the gating strategy. Representative histograms show hCD59 expression.

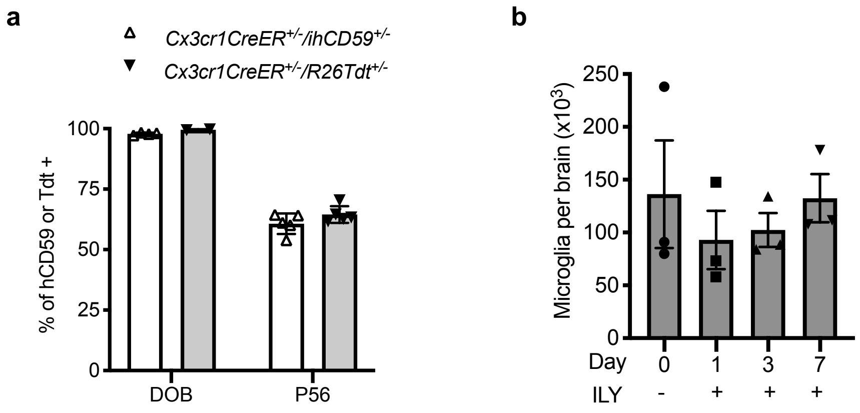

Supplementary Figure 3. E18.5 Tamoxifen labelling hCD59 on RMs (a) and the necrotic effect of ILY on microglia (b). (**a**) The percentage of hCD59 or Tdt+ RMs within total RMs after E18.5 Tam labelling.(mean ± s.e.m., *Cx3cr1CreER* $^{+/-}$*/ihCD59*$^{+/-}$ group: n=4 for DOB and n=5 for P56; *Cx3cr1CreER* $^{+/-}$*/R26tdt*$^{+/-}$ group: n=2 for DOB and n=5 for P56). (**b**) Microglia cell counts from *Cx3cr1CreER* $^{+/-}$*/ihCD59*$^{+/-}$ mice without ILY (D0) or at different days (1, 3 and 7) after ILY injection. (mean ± s.e.m., n=3 mice per time point per group). Source data are provided as a Source Data file.

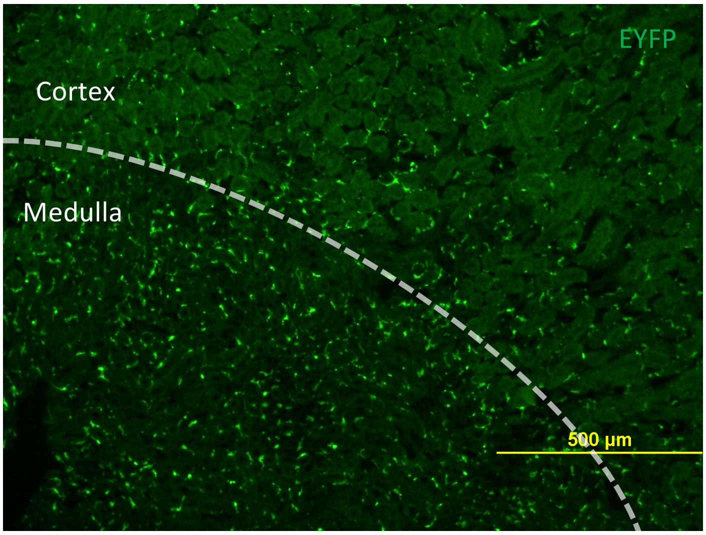

Supplementary Figure 4. Immunofluorescence (IF) staining of EYFP in kidney of adult *Cx3cr1CreER*$^{+/-}$ mice. 10x magnification. White dash line are used to define cortex and medulla based on tissue morphology. Image is representative of three independent staining from three mice.

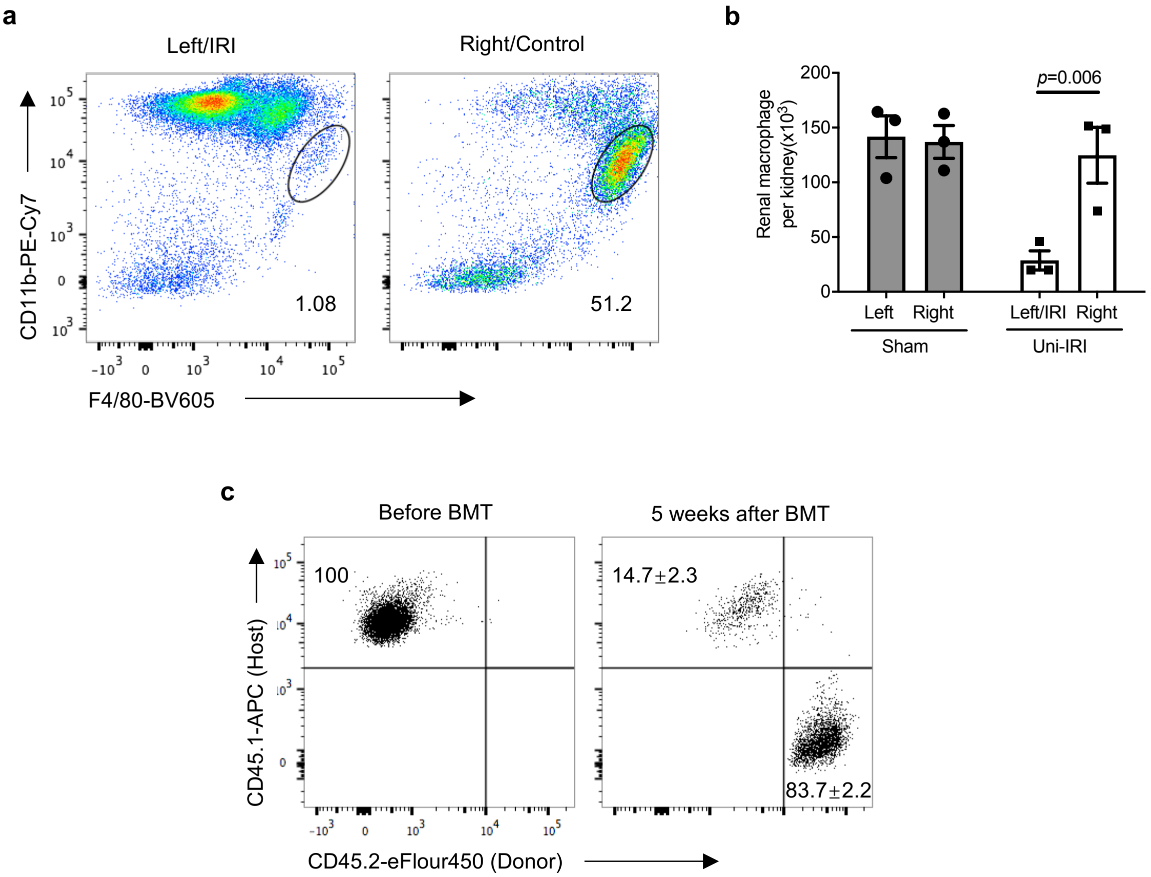

Supplementary Figure 5. The effect of ischemia-reperfusion injury (IRI) and bone marrow transplantation (BMT) on RMs. (**a**,**b**) Unilateral renal ischemia-reperfusion was performed in WT mice for 30 min. (**a**) Representative flow cytometry plots of RMs after ischemia-reperfusion injury(IRI). Left plot is injured kidney, right plot is sham control from same mice. (**b**) Cell counts of RMs. Sham is non-surgery control mice. (mean ± s.e.m., two-tailed unpaired t-test, n=3 mice per group). (**c**) Chimerism of CD45.1 and CD45.2 in RMs without BMT (Left) or 5 weeks after BMT (Right). (mean ± s.e.m., n=4 mice per group). Source data are provided as a Source Data file.

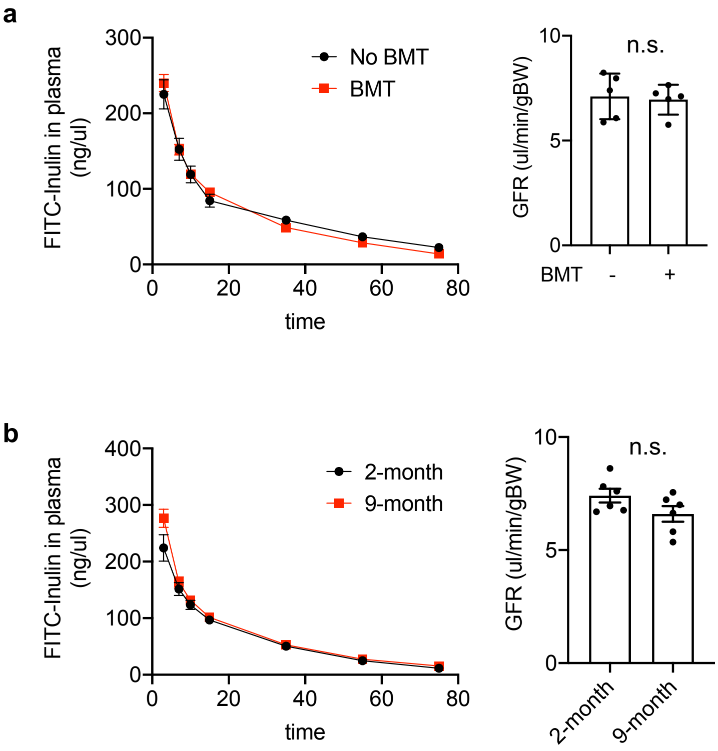

Supplementary Figure 6. The effect of the irradiation and aging on kidney function. (**a**) Glomerular filtration rate (GFR) measurements in male *Cx3cr1CreER*$^{+/-}$*/ihCD59*$^{+/-}$ mice at 2-month after bone marrow transplantation (BMT) or age-matched control (No BMT) (mean ± s.e.m., n=5 mice per group). (**b**) GFR measurements in 2-month and 9-month old male *Cx3cr1CreER*$^{+/-}$*/ihCD59*$^{+/-}$ mice (mean ± s.e.m., n=6 mice per group). Two-tailed unpaired t-test. n.s. indicates $p > 0.05$. Left: plasma clearance kinetics of FITC-inulin; Right: summarized GFR results showed in respective left panel. Source data are provided as a Source Data file.

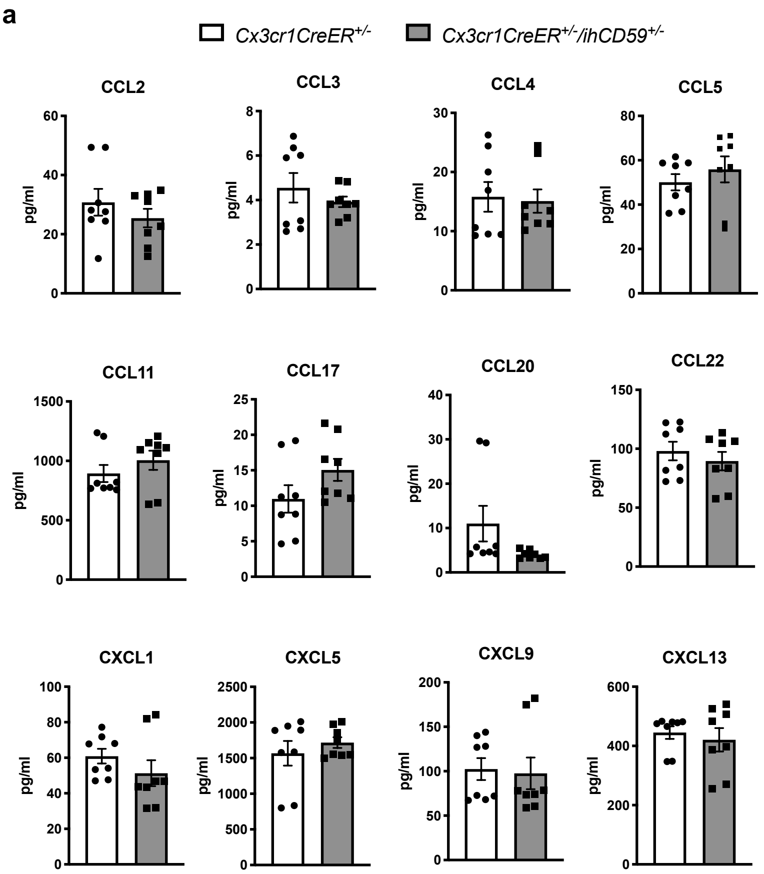

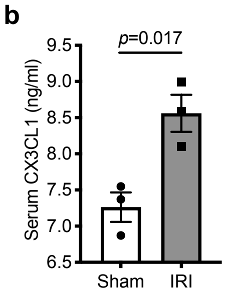

Supplementary Figure 7. Serum chemokine levels after RMs depletion. (**a**) Serum chemokine levels at 1 day after ILY-mediated RMs depletion. (mean ± s.e.m., n=8 mice per group). (B) Serum Cx3CL1 level 18h after IRI in mice. (mean ± s.e.m., two-tailed unpaired t-test, n=3 mice per group). Source data are provided as a Source Data file.

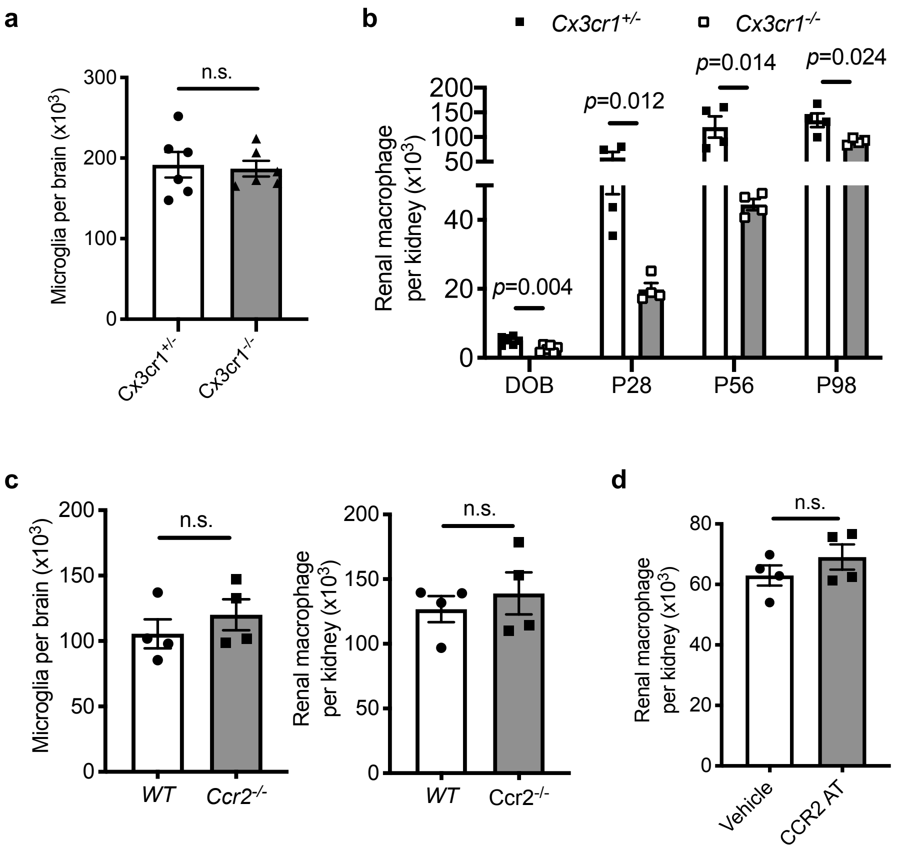

Supplementary Figure 8. The effect of CX3CR1 or CCR2 deficiency/antagonism on RMs and microglia. (**a**) Microglia cell counts in 2-month old *Cx3cr1*$^{+/-}$ and *Cx3cr1*$^{-/-}$ mice. (n=6 mice per group). (**b**) RMs cell counts in *Cx3cr1*$^{+/-}$ and *Cx3cr1*$^{-/-}$ mice from DOB to P98. (n=6 mice for DOB, n=4 mice for P28,56,98). (**c**) Microglia (left) and RMs(right) cell counts in 2-month old *WT* and *Ccr2*$^{-/-}$ mice. (n=4 mice per group). (**d**) RMs cell counts at 7 days after ILY injection followed by daily CCR2 antagonism or DMSO (Vehicle) treatment. (n=4 mice per group). All data are presented as mean ± s.e.m.. *p*-Values by two-tailed unpaired t-test are indicated in (a,b,c,d). n.s. indicates $p > 0.05$ Source data are provided as a Source Data file.

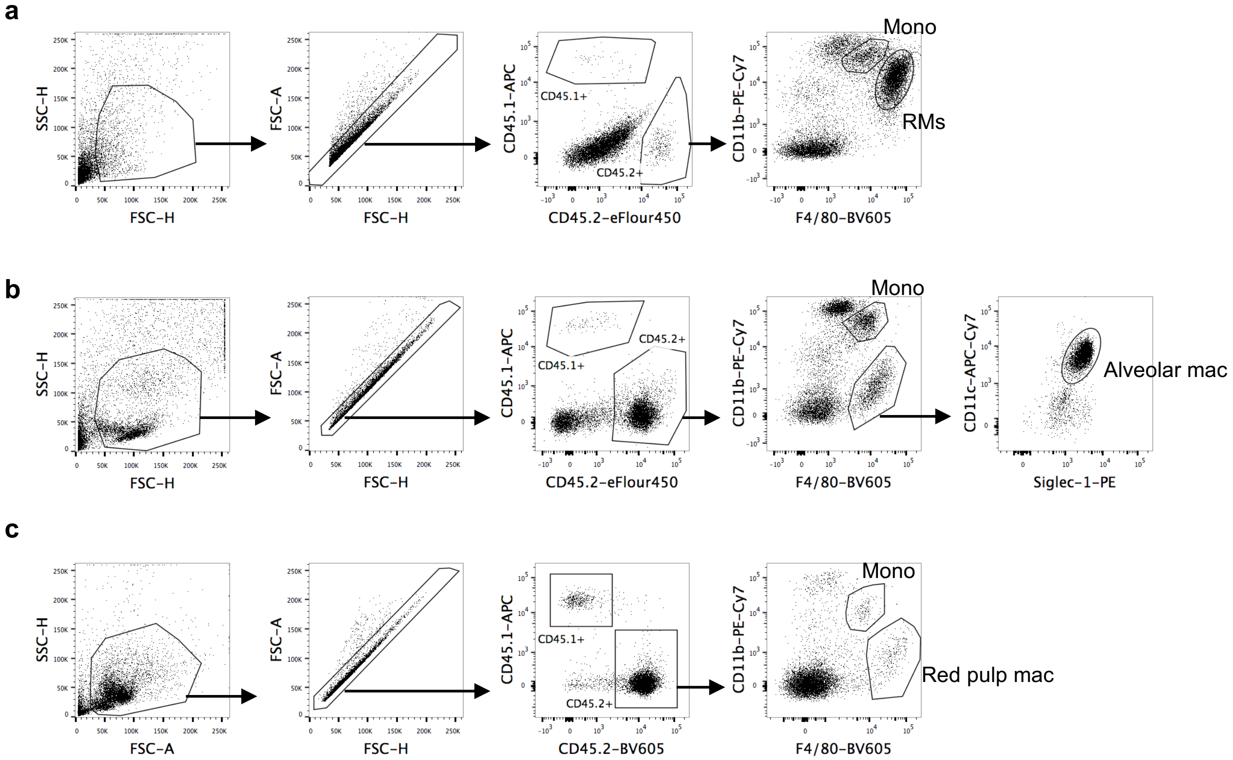

Supplementary Figure 9. Gating strategy of monocytes (Mono) and macrophages (mac) in kidney (a), lung (b).and spleen (c) of bone marrow transplanted mice in fig. 5d. (**a**) RMs (CD45.2^{+}CD11b^{+}F4/80^{high}), mono (CD45.2^{+}CD11b^{+}F4/80^{low}). (**b**) Lung alveola mac (CD45.2^{+}CD11b^{low}F4/80^{high}CD11c^{+}Siglec-1^{+}), mono (CD45.2^{+}CD11b^{+}F4/80^{low}). (**c**) Spleen red pulp mac (CD45.2^{+}CD11b^{low}F4/80^{high}), mono (CD45.2^{+}CD11b^{+}F4/80^{low}).

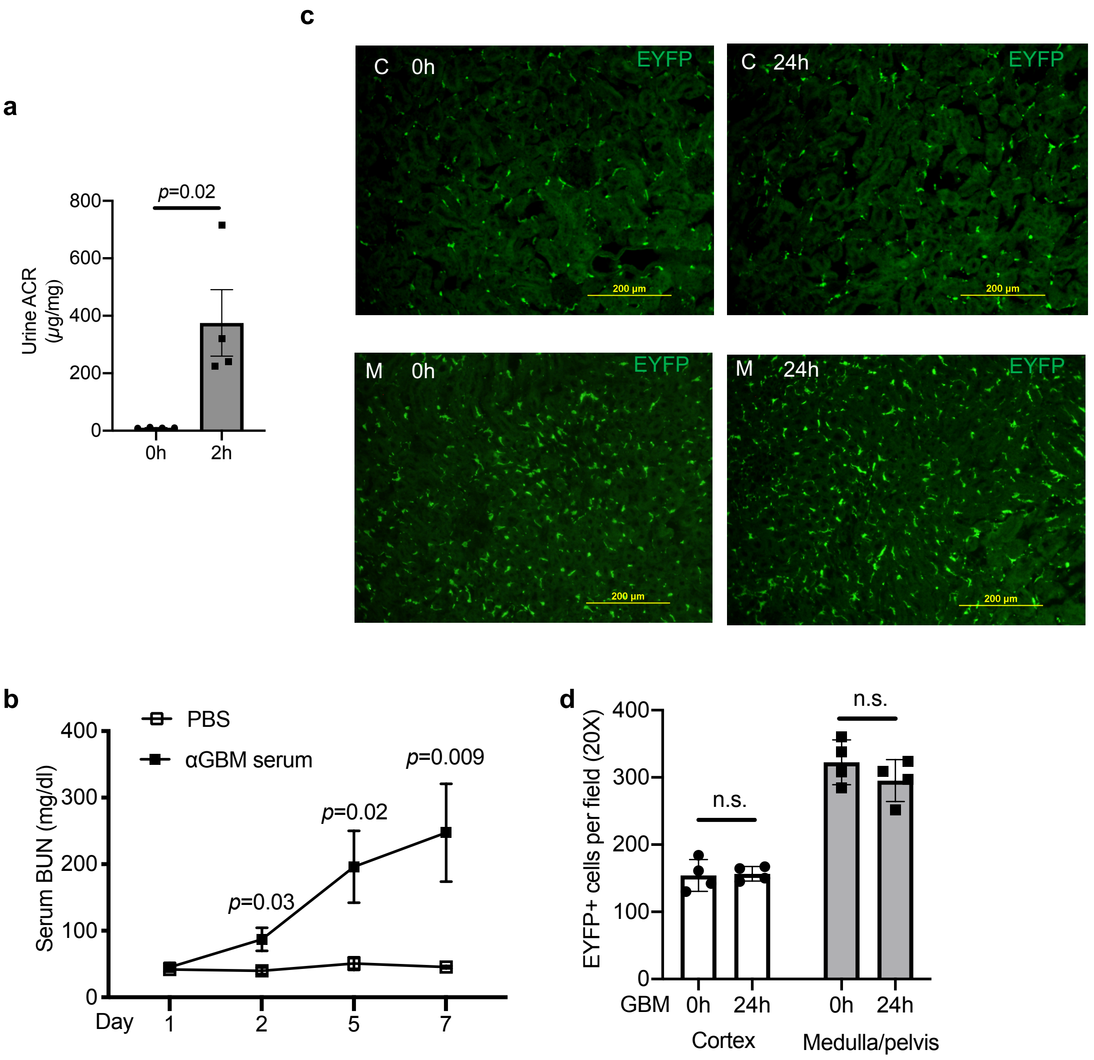

Supplementary Figure 10. Urine albumin-to-creatinine ratio (ACR)/blood urine nitrogen (BUN) levels and kidney EYFP staining after αGBM serum injection. (**a**) Urine ACR level at 2h after αGBM serum injection (n=4 mice per time point). (**b**) Serum BUN level at different days after αGBM serum or PBS injection (n=4 mice per time point per group). (**c**) IF staining of EYFP and (**d**) quantification of EYFP+ cells in cortex (C) and medulla/pelvis (M) from αGBM serum treated mice. Each dot represents average cell numbers from one mice (n=4 mice per time point). All data are presented as mean ± s.e.m.. *p*-Values by two-tailed unpaired t-test are indicated in (a,b,d), n.s. indicates $p > 0.05$. Source data are provided as a Source Data file.

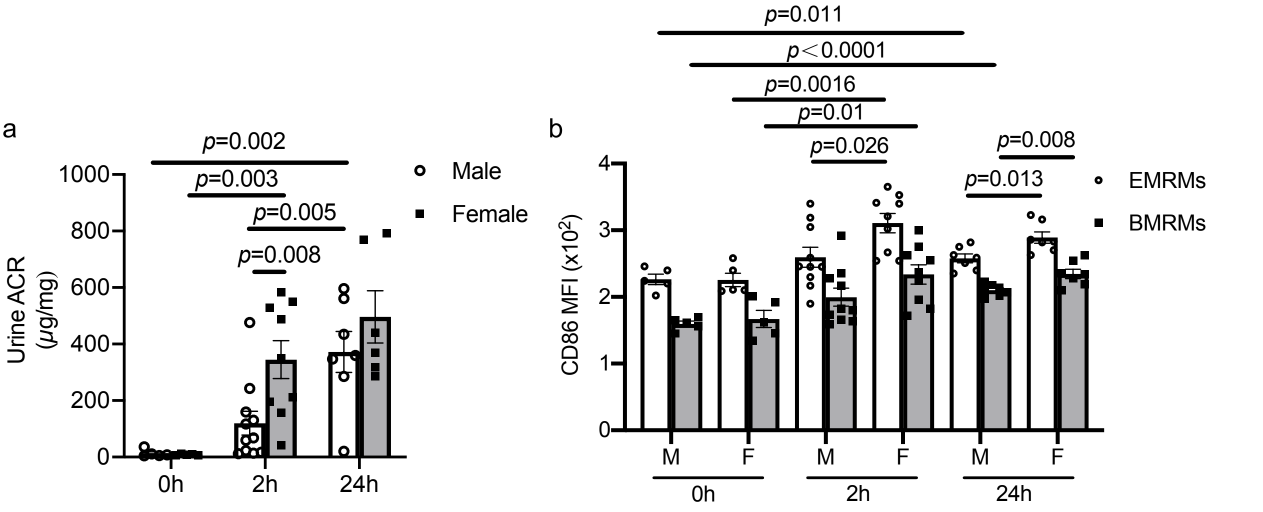

Supplementary Figure 11. Gender differences in RMs response to αGBM-induced nephritis. (**a**) Urine ACR level at 2h and 24h after αGBM serum injection in male or female *Cx3cr1CreER* $^{+/-}$*/ihCD59*$^{+/-}$ mice. (mean ± s.e.m., 0h: n=5 per group; 2h: n=11 male and n=9 female; 24h: n=7 male and n=6 female). (**b**) MFI of CD86 expression in EMRMs and BMRMs in male or female *Cx3cr1CreER* $^{+/-}$*/ihCD59*$^{+/-}$ mice. 0h indicates no-αGBM treated mice. (mean ± s.e.m., 0h: n=5 male and female; 2h: n=10 male and n=9 female; 24h: n=7 male and female). *p*-Values by two-tailed unpaired t-test are indicated in (a,b). Source data are provided as a Source Data file.

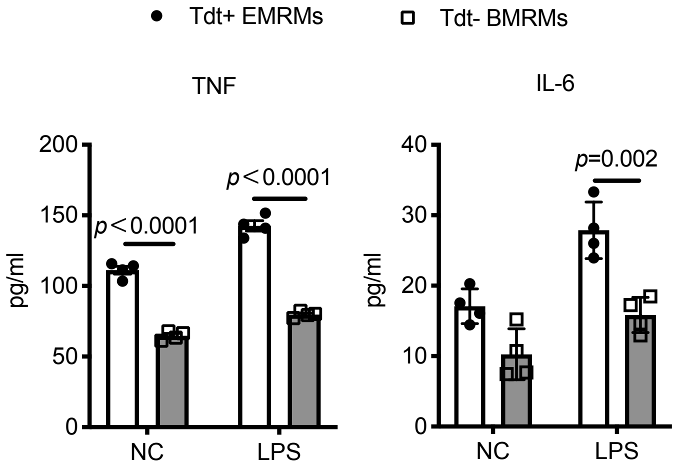

Supplementary Figure 12. **TNF and IL-6 production after LPS treatment in Tdt+ EMRMs and Tdt- BMRMs**. TNF and IL-6 level in the supernatant of sorted Tdt+ EMRMs and Tdt- BMRMs treated with medium (NC) or 100ng/ml LPS for 18h. Data are pooled from two independent experiments and each dot represents cells obtained from individual sorting. (mean ± s.e.m., two-tailed unpaired t-test, n=4 per group). Source data are provided as a Source Data file.

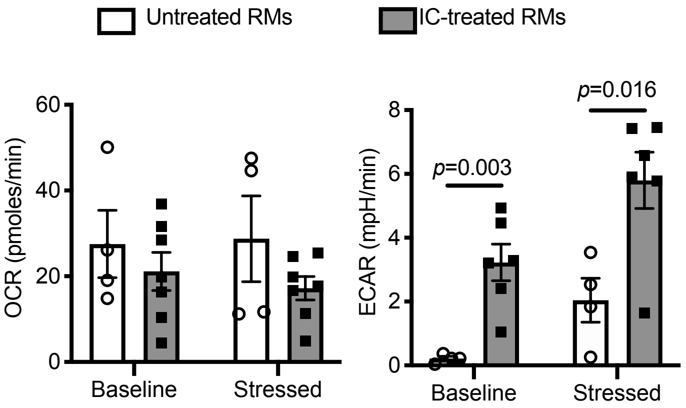

Supplementary Figure 13. Energy phenotype test in RMs after Immune complex (IC) stimulation. Quantification of the OCR(left) and ECAR(right) in hCD59+ EMRMs and hCD59- BMRMs sorted from untreated or IC-treated *Cx3cr1CreER* $^{+/-}$*/ihCD59*$^{+/-}$ mice. Data are pooled from two independent experiments (mean ± s.e.m., two-tailed unpaired t-test, OCR: n=4 for untreated and n=7 for IC-treated; ECAR:n=4 for untreated and n=6 for IC-treated). Source data are provided as a Source Data file.

Supplementary Table 1. Antibodies for flow cytometry

Antibody	Clone	Cat Number	Company
CD45	30-F11	48-0451-82	eBioscience
CD45.1	A20	17-0453-81	eBioscience
CD45.2	104	48-0454-82	eBioscience
CD11b	BM8	17-4801-80	eBioscience
CD11c	N418	47-0114-82	eBioscience
F4/80	BM8	123133	Biolegend
CX3CR1	SA011F11	149006	Biolegend
CX3CR1	SA011F11	149019	Biolegend
I-A/I-E	M5/114.15.2	107607	Biolegend
Ki67	SolA15	12-5698-80	eBioscience
Ki67	SolA15	17-5698-82	eBioscience
hCD59	OV9A2	17-0596-42	eBioscience
hCD59	OV9A2	12-0596-42	eBioscience
Ly6C	HK1.4	48-5932-80	eBioscience
Ly6G	1A8	127613	Biolegend
Purified CD16/32	93	14-0161-85	eBioscience
CD64 (FcγRI)	X54-5/7.1	139306	Biolegend
CD16/32(FcγRII/III)	93	48-0161-80	ebioscience
CD16.2 (FcγRIV)	9E	149505	Biolegend
CCR2	SA203G11	150628	Biolegend
CD86	GL-1	105011	Biolegend
TNF	MP6-XT22	17-7321-81	eBioscience
TNF	MP6-XT22	12-7321-41	eBioscience
CD169 (Siglec-1)	3D6.112	142403	Biolegend
