# Supplementary material for: Distinct fate, dynamics and niches of renal macrophages of bone marrow or embryonic origins
Source: Nat Commun. 2020 May 8;11:2280. doi: 10.1038/s41467-020-16158-z (PMC7210253; doi:10.1038/s41467-020-16158-z)
Supplement: Supplementary file 3 — Reporting Summary [file 41467_2020_16158_MOESM3_ESM.pdf]

nature research

Corresponding author(s): Xuebin Qin

Last updated by author(s): Apr 9, 2020

Reporting Summary

Nature Research wishes to improve the reproducibility of the work that we publish. This form provides structure for consistency and transparency in reporting. For further information on Nature Research policies, see Authors & Referees and the Editorial Policy Checklist.

Statistics

For all statistical analyses, confirm that the following items are present in the figure legend, table legend, main text, or Methods section.

n/a	Confirmed	
☐	☒	The exact sample size (*n*) for each experimental group/condition, given as a discrete number and unit of measurement
☐	☒	A statement on whether measurements were taken from distinct samples or whether the same sample was measured repeatedly
☐	☒	The statistical test(s) used AND whether they are one- or two-sided<br>*Only common tests should be described solely by name; describe more complex techniques in the Methods section.*
☒	☐	A description of all covariates tested
☒	☐	A description of any assumptions or corrections, such as tests of normality and adjustment for multiple comparisons
☐	☒	A full description of the statistical parameters including central tendency (e.g. means) or other basic estimates (e.g. regression coefficient) AND variation (e.g. standard deviation) or associated estimates of uncertainty (e.g. confidence intervals)
☐	☒	For null hypothesis testing, the test statistic (e.g. *F*, *t*, *r*) with confidence intervals, effect sizes, degrees of freedom and *P* value noted<br>*Give P values as exact values whenever suitable.*
☒	☐	For Bayesian analysis, information on the choice of priors and Markov chain Monte Carlo settings
☒	☐	For hierarchical and complex designs, identification of the appropriate level for tests and full reporting of outcomes
☒	☐	Estimates of effect sizes (e.g. Cohen's *d*, Pearson's *r*), indicating how they were calculated

Our web collection on statistics for biologists contains articles on many of the points above.

Software and code

Policy information about availability of computer code

Data collection	FACSDiva v6.1
Data analysis	Flowjo 10. Graphpad Prism 8

For manuscripts utilizing custom algorithms or software that are central to the research but not yet described in published literature, software must be made available to editors/reviewers. We strongly encourage code deposition in a community repository (e.g. GitHub). See the Nature Research guidelines for submitting code & software for further information.

Data

Policy information about availability of data

All manuscripts must include a data availability statement. This statement should provide the following information, where applicable:

- Accession codes, unique identifiers, or web links for publicly available datasets
- A list of figures that have associated raw data
- A description of any restrictions on data availability

The source data underlying Figs.1b-d, 2b-f, 3b,d, 4b,c,e,g,h, 5a-c,e,f, 6c-f, 7a-g, 8a-e and Supplementary Figs. 3a,b, 5b,c, 6a,b, 7a,b, 8a-d,10-13 are provided as a Source Data file. All other data are available from the corresponding authors upon reasonable requests.

Field-specific reporting

Please select the one below that is the best fit for your research. If you are not sure, read the appropriate sections before making your selection.

☒ Life sciences ☐ Behavioural & social sciences ☐ Ecological, evolutionary & environmental sciences

Life sciences study design

All studies must disclose on these points even when the disclosure is negative.

Sample size	Due to the limited number of animals from per litter, no statistical methods were used to determine sample size. The size was chosen depending on the our previous publication (Cre-inducible human CD59 mediates rapid cell ablation after intermedilysin administration. J Clin Invest, 2016. 126(6): p. 2321-33). We repeated the experiments at lease two times to make sure the reproducibility of the data.
Data exclusions	No data was excluded from the analysis
Replication	Experiments were successfully performed a minimum of two times and/or with sufficient animals per group to demonstrate statistical significance.
Randomization	Mice were randomly divided into different group and the body weight, age were similar between groups
Blinding	Investigators are blinded to group allocation in figure 3,4, 5 and 7 . In the experiment comparing mice at different age such as figure 1 and 2, blinding was not possible. In other experiments, non-blinding will not influence since the analysis was performed in same batch. In Fig.3, IF quantification was done by individual who is blinded to the experimental design

Reporting for specific materials, systems and methods

We require information from authors about some types of materials, experimental systems and methods used in many studies. Here, indicate whether each material, system or method listed is relevant to your study. If you are not sure if a list item applies to your research, read the appropriate section before selecting a response.

Materials & experimental systems

n/a	Involved in the study	
☐	☒	Antibodies
☒	☐	Eukaryotic cell lines
☒	☐	Palaeontology
☐	☒	Animals and other organisms
☒	☐	Human research participants
☒	☐	Clinical data

Methods

n/a	Involved in the study	
☒	☐	ChIP-seq
☐	☒	Flow cytometry
☒	☐	MRI-based neuroimaging

Antibodies

Antibodies used

Antibodies were used for flow cytometry and immunofluorescence staining
Flow cytometry: 1:100 dilution was used in general
CD45-eFlour450 30-F11 48-0451-82 eBioscience
CD45.1-APC A20 17-0453-81 eBioscience
CD45.2-eFlour450 104 48-0454-82 eBioscience
CD11b-PE-Cy7 BM8 17-4801-80 eBioscience
CD11c-APC-Cy7 N418 47-0114-82 eBioscience
F4/80-BV605 BM8 123133 Biolegend
CX3CR1-PE SA011F11 149006 Biolegend
CX3CR1-FITC SA011F11 149019 Biolegend
I-A/I-E-APC M5/114.15.2 107607 Biolegend
Ki67-PE SolA15 12-5698-80 eBioscience
Ki67-APC SolA15 17-5698-82 eBioscience
hCD59-APC OV9A2 17-0596-42 eBioscience
hCD59-PE OV9A2 12-0596-42 eBioscience
Ly6C-eFlour450 HK1.4 48-5932-80 eBioscience
Ly6G-APC 1A8 127613 Biolegend
Purified CD16/32 93 14-0161-85 eBioscience
CD64-APC (FcγRI) X54-5/7.1 139306 Biolegend
CD16/32-APC (FcγRII/III) 93 48-0161-80 ebioscience
CD16.2-APC (FcγRIV) 9E 149505 Biolegend
CCR2-APC SA203G11 150628 Biolegend
CD86-APC GL-1 105011 Biolegend
TNF-APC MP6-XT22 17-7321-81 eBioscience
TNF-PE MP6-XT22 12-7321-41 eBioscience
CD169-PE (Siglec-1) 3D6.112 142403 Biolegend
immunofluorescence staining and others, working concentration is listed in the manuscript
rabbit anti-GFP antibody (A-11122, Invitrogen)
Goat anti-Rabbit IgG (H+L) Cross-Adsorbed Secondary Antibody, Alexa Fluor 488 (A-11008, Invitrogen)

rabbit anti-BSA IgG (A11133, Invitrogen)

Validation

According to vender's data sheets, all antibodies used in the manuscript has been pre-validated by the manufacturer and reported by other researcher to work for flow cytometry or immunofluorescence staining in mice.

Animals and other organisms

Policy information about studies involving animals; ARRIVE guidelines recommended for reporting animal research

Laboratory animals

All mice are in C57BL/6 background in the study. CD45.1 C57BL/6 (JAX 002014), CD45.2 C57BL/6 (JAX 000664), Cx3cr1CreER+/+ (JAX 021160), Ccr2 -/- (JAX 004999), R26-Tdto- mato +/+ (JAX 007905) mice were purchased from The Jackson Laboratory (Bar Harbor, ME).The ihCD59+/+ mice were previously generated by our lab and backcrossed with C57BL/6 background for at least seven generations. Mice used in the study is from newborn to 62 weeks old. Male mice were used for bone marrow transplantation, glomerular filtration rate and LPS-induced kidney injury. Both male and female were used in other experiments. All animal experiments were reviewed and approved prior to commencement of the activity by the Animal Care and Use Committee from Tulane University (permit numbers 633 and 638) and Temple University (permit numbers 4598 and 4738). Mice were housed in the animal facility of Tulane University School of Medicine or Lewis Katz School of Medicine at Temple University, with a 12-h light/dark cycle, in specific-pathogen-free environment.

Wild animals

The study did not involve wild animals

Field-collected samples

The study did not involve samples collected from the field

Ethics oversight

Animal studies were approved by the Animal Care and Use Committee from the Temple University Lewis Katz School of Medicine and Tulane University School of Medicine

Note that full information on the approval of the study protocol must also be provided in the manuscript.

Flow Cytometry

Plots

Confirm that:

☒ The axis labels state the marker and fluorochrome used (e.g. CD4-FITC).

☒ The axis scales are clearly visible. Include numbers along axes only for bottom left plot of group (a 'group' is an analysis of identical markers).

☒ All plots are contour plots with outliers or pseudocolor plots.

☒ A numerical value for number of cells or percentage (with statistics) is provided.

Methodology

Sample preparation

Sample preparation was provided in the Methods. The Brain, lung, heart, and kidney were collected from PBS-perfused mice and mechanically dissociated and enzymatic digested, followed by percoll centrifugation. The blood was collected from mice eye. The peritoneum was isolated from mice abdomen. BM single cells were flushed out from femurs. Spleen and liver were mashed with plungers of 5ml syringe in PBS. Cells were passed through 70 um cell strainers and stained with antibody

Instrument

BD LSRII; BD AriaIIu

Software

FACSDiva is used for data collection and Flowjo 10 is used for data analysis

Cell population abundance

Abundance is above 90% among post-sorting fraction determined by second round of flow cytometry analysis

Gating strategy

Gating strategy was provided in supplemental figure 1 and 9. The FSC/SSC gates include all cell population exclude debris depending on the size. Fluorescence minus one or isotype staining were used to discriminate positive and negative staining.

☒ Tick this box to confirm that a figure exemplifying the gating strategy is provided in the Supplementary Information.
